# Poster Session II - A231 AZATHIOPRINE DOSE THRESHOLD IN ANTI TNF COMBINATION THERAPY FOR MINIMIZING IMMUNOGENICITY IN IBD: A RETROSPECTIVE COHORT STUDY

**DOI:** 10.1093/jcag/gwaf042.231

**Published:** 2026-02-13

**Authors:** N Alajeel, J Choi, R Khanna, A Wilson

**Affiliations:** Internal Medicine, London Health Sciences Centre, London, ON, Canada; Internal Medicine, London Health Sciences Centre, London, ON, Canada; Internal Medicine, London Health Sciences Centre, London, ON, Canada; Internal Medicine, London Health Sciences Centre, London, ON, Canada

## Abstract

**Background:**

Prior studies suggest similar outcomes with low (<2 mg/kg/day) versus standard (≥2 mg/kg/day) azathioprine (AZA) when combined with TNF α inhibitors (TNFi), but many did not account for pharmacogenetics. Thiopurine methyltransferase (*TPMT*) and HLA DQA1*05G>A variants influence AZA exposure and anti drug antibody (ADA) risk, potentially confounding apparent dose effects.

**Aims:**

To compare low versus standard dose AZA in IBD patients on combination TNFi for ADA and key clinical outcomes, while adjusting for clinical and genetic covariates.

**Methods:**

Single center retrospective cohort of adults with IBD receiving AZA plus infliximab or adalimumab from induction through maintenance. Only participants with a wildtype *TPMT* status were included in the adjusted analyses. Groups: low dose (<2 mg/kg/day) vs standard dose (≥2 mg/kg/day). The primary outcome ADA formation. Secondary outcomes included TNFi discontinuation, loss of response (LOR), and adverse drug events (ADEs). Multivariable analysis, reported as Odd ratios or hazard ratios, where appropriate, were adjusted for age, sex, IBD type, TNFi type, and HLA DQA1*05G>A genotype. Receiver operating characteristic (ROC) analysis tested for a weight based AZA threshold associated with ADA formation.

**Results:**

A total of 119 participants (low n = 68; standard n = 51) are included in the analyses. There were no differences in baseline characteristics between participants receiving low versus standard dose AZA. Median follow up time was 35 months. Adjusted analyses in *TPMT* wildtype participants showed no increased TNFi ADA risk with standard vs low dose AZA (HR 1.18; 95% CI 0.78–1.81; p = 0.44). Ther was no difference in the incidence of TNFi discontinuation (HR 0.68; 0.31–1.50; p = 0.34), LOR (OR 1.40; 0.61–3.32; p = 0.44), or ADEs (OR 0.93; 0.37–2.40; p = 0.87). ROC analysis did not identify a discriminative weight based threshold for ADA formation (AUC 0.53; 95% CI 0.39–0.67; p = 0.65).

**Conclusions:**

To date, low dose AZA performed comparably to standard dose AZA across ADA, discontinuation, LOR, and ADEs after adjustment for HLA DQA1*05G>A genotype and clinical factors in participants receiving combination TNFi therapy for IBD. No clinically useful weight based AZA threshold for ADA was identified, supporting flexible AZA dosing in TPMT wildtype patients.

A231 Table 1: Univariate analyses

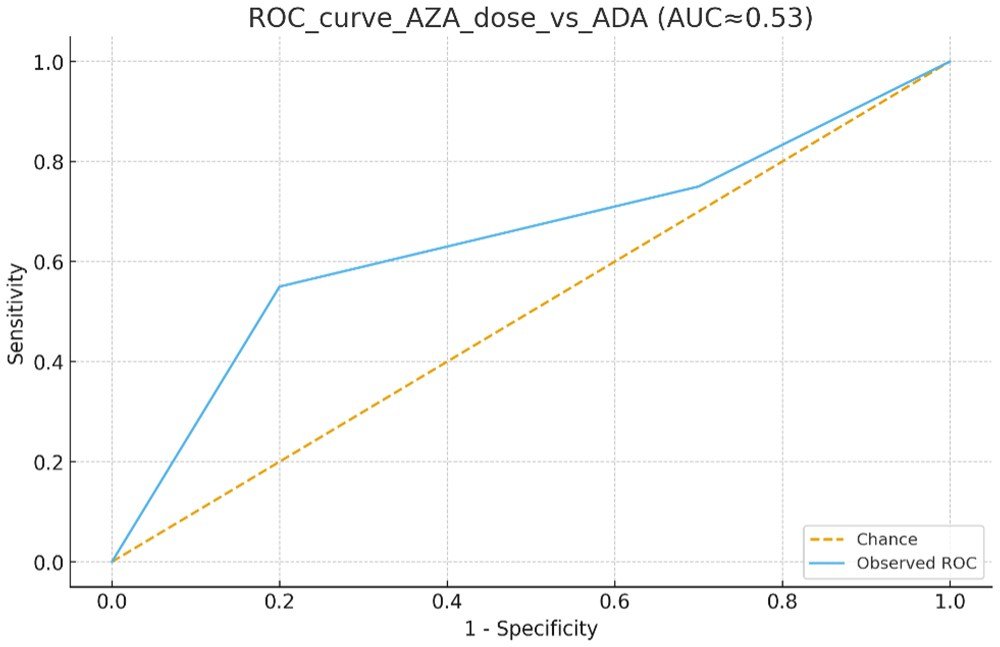

**Funding Agencies:**

None

**Table:** 

Outcome	Standard dose AZA (n = 51)	Low dose AZA (n = 68)	Unadjusted p-value
Antidrug antibodies	12 (24%)	9 (13%)	0.16
TNFA discontinuation	13 (25.5%)	22 (32%)	0.54
TNFA loss of response	12 (23.5%)	21 (30.9%)	0.41
Adverse drug event	12 (23.5%)	14 (20.6%)	0.82

